# Simultaneous hydrolysis of carbaryl and chlorpyrifos by *Stenotrophomonas* sp. strain YC-1 with surface-displayed carbaryl hydrolase

**DOI:** 10.1038/s41598-017-13788-0

**Published:** 2017-10-17

**Authors:** Chao Yang, Xiaoqing Xu, Yanping Liu, Hong Jiang, Yunbo Wu, Ping Xu, Ruihua Liu

**Affiliations:** 1 College of Life Sciences, Nankai University, Tianjin, 300071 China; 2 Department of Gynaecology and Obstetrics, Tianjin Medical University General Hospital, Tianjin, 300052 China; 3 Institute of Zoology, Chinese Academy of Sciences, Beijing, 100101 China; 4 State Key Laboratory of Microbial Metabolism, School of Life Sciences and Biotechnology, Shanghai Jiao Tong University, Shanghai, 200240 China

## Abstract

Many sites are often co-contaminated with multiple pesticides. To date, there are no reports on simultaneous degradation of different classes of pesticides by a natural microorganism. In this work, we aim at constructing a live biocatalyst able to simultaneously hydrolyze carbaryl and chlorpyrifos. For this purpose, carbaryl hydrolase (CH) was displayed on the cell surface of a chlorpyrifos-degrading bacterium *Stenotrophomonas* sp. strain YC-1 using N- and C-terminal domain of ice nucleation protein (INPNC) from *Pseudomonas syringae* INA5 as an anchoring motif. The localization of INPNC-CH fusion protein in the outer membrane fraction was demonstrated by cell fractionation followed by Western blot analysis. Surface display of INPNC-CH was further confirmed by proteinase accessibility experiment and immunofluorescence microscope. CH was present in an active form on cell surface without causing any growth inhibition, suggesting that the INP-based display system is a useful tool for surface expression of macromolecular heterologous proteins on the bacterial cell surface. Because surface-displayed CH has free access to pesticides, this bacterium can be used as a whole-cell biocatalyst for efficient hydrolysis of pesticides.

## Introduction

Chlorpyrifos is a moderately toxic organophosphorus pesticide that has played an important role in controlling major agricultural pests and raising agricultural productivity. Chlorpyrifos leads to the loss of nerve function of vertebrates by irreversibly inhibiting acetylcholine esterase (AChE) in the central nervous system^1,2^. Carbaryl is a broad-spectrum carbamate insecticide and is a potent AChE inhibitor^3,4^. Carbaryl and chlorpyrifos are often used simultaneously to control agricultural pests; thus, many sites are co-contaminated with carbaryl and chlorpyrifos. Especially, the co-existence of different classes of pesticides can potentiate the toxicity of individual pesticides and the joint toxicity of pesticides poses a great threat to human health^5^.

Microorganisms have evolved degradation pathways for the removal of synthesized pesticides. So far, several carbaryl-degrading bacteria, such as *Rhizobium* sp. strain AC100^6^, *Arthrobacter* sp. strain RC100^4,7^, *Blastobacter* sp. strain M501^3^, and *Pseudomonas* sp. strains C4, C5, and C6^8–10^, have been isolated. Moreover, a carbaryl hydrolase gene (*cehA*) was cloned from *Rhizobium* sp. strain AC100^6^. To date, several chlorpyrifos-degrading bacteria, such as *Enterobacter* strain B-14^11^, *Alcaligenes faecalis* strain DSP3^12^, *Stenotrophomonas* sp. strain YC-1^13^, *Sphingomonas* sp. strain Dsp-2^14^, *Paracoccus* sp. strain TRP^15^, *Bacillus pumilus* strain C2A1^16^, and *Cupriavidus* sp. strain DT-1^17^, have been isolated by selective enrichment with chlorpyrifos. Unfortunately, microorganisms with the capability to simultaneously degrade chlorpyrifos and carbaryl have not yet been isolated from the environment. Therefore, the construction of recombinant microorganisms is useful for the remediation of multiple pesticides-contaminated sites.

Carbaryl-degrading bacteria have been shown to produce intracellular carbaryl hydrolase (CH), and therefore intracellular CH cannot directly bind to extracellular substrates^3,6,7^. This bottleneck, however, could be eliminated if CH is displayed onto the surface of cells. Previous studies have shown that surface-displayed organophosphorus hydrolase (OPH) can directly interact with substrates without the membrane permeability barrier, thereby improving the overall catalytic efficiency^18–20^.

Various surface-anchoring motifs, such as Lpp-OmpA chimera, ice nucleation protein (INP), and autotransporter, have been widely used to display proteins on the surface of bacterial cell^21,22^. INP, an outer membrane protein found in *Pseudomonas syringae*, *Xanthomonas campestris*, and *Erwinia herbicola*, accelerates the formation of ice crystal in supercooled water. INP is composed of three distinct domains, including an N-terminal domain, a C-terminal domain, and a highly repetitive central domain^23^. INP-mediated cell surface display can be achieved using either full-length INP or truncated INP through C-terminal fusion^18,24,25^. Various types of INPs (e.g., InaK, InaPb, InaQ, InaV and InaZ) have been successfully used to display heterologous proteins, such as levansucrase^24^, synthetic phytochelatins^26^, OPH^18–20,27^, green fluorescent protein (GFP)^25^, chitinase^28^, NADPH-cytochrome P450 oxidoreductase (CPR)^29^, human poliovirus receptor^30^, carbonic anhydrase^31^, glucose dehydrogenase^32^, glutamate dehydrogenase^33^, xylose dehydrogenase^34,35^, *mycoplasma* adhesion proteins^36^, human norovirus capsid proteins^37^, formate dehydrogenase^38^, and human arginase-1^39^, on the bacterial cell surface.

We previously isolated a chlorpyrifos-degrading bacterium *Stenotrophomonas* sp. strain YC-1, and this strain could utilize chlorpyrifos as the sole source of carbon for growth. Moreover, a chromosome-based *mpd* gene coding for chlorpyrifos hydrolase (CPH) was cloned from strain YC-1^13^. CH and CPH require no cofactors for their activity. In this work, CH encoded by the *cehA* gene (GenBank accession no. AB069723) from *Rhizobium* sp. strain AC100 was functionally displayed on the cell surface of *Stenotrophomonas* sp. strain YC-1 using the truncated InaV (GenBank accession no. AJ001086) from *P*. *syringae* INA5 as an anchoring motif, resulting in a recombinant strain capable of simultaneously hydrolyzing carbaryl and chlorpyrifos.

## Results and Discussion

### Surface localization of INPNC-CH fusion protein

In this study, the feasibility of targeting CH onto the cell surface of *Stenotrophomonas* sp. strain YC-1 was investigated using the N- and C-terminal domain of InaV (INPNC)^40^ as an anchoring motif. To create the surface expression vector pVIC3, the *inpnc-cehA* fusion gene was subcloned into a medium-copy-number vector, pVLT33. The broad-host-range vector, pVLT33, has an ability to replicate in a wide variety of Gram-negative bacteria because it is an RSF1010 derivative^41^. Expression of INPNC-CH was tightly regulated by an inducible *tac* promoter and the *lacI* ^q^ gene on the plasmid pVLT33.

**Figure 1:**
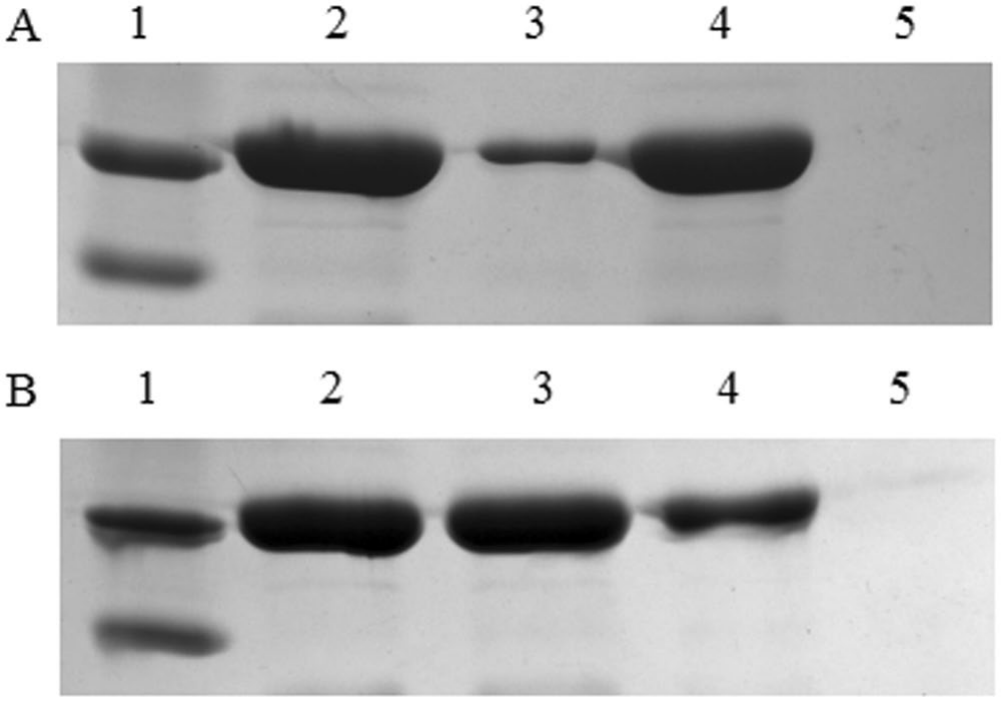
Western blot analysis for subcellular localization of INPNC-CH fusion protein in *Stenotrophomonas* sp. strain YC-1/pVIC3 (**A**) and *E. coli* DH5α/pVIC3 (**B**). Lane 1, protein marker; lane 2, whole-cell lysates; lane 3, soluble fraction; lane 4, outer membrane fraction; lane 5, whole-cell lysates of YC-1/pVLT33 (or DH5α/pVLT33).

To verify whether INPNC-CH fusion protein was produced in the recombinant strain YC-1 carrying pVIC3, Western blot analysis was performed using rabbit polyclonal anti-CH antibodies. As a result, a specific immunoreactive band appeared at the position of ~120 kDa was detected in whole-cell lysates of YC-1 cells carrying pVIC3 (Fig. 1A, lane 2), which matches well with the molecular weight of INPNC-CH fusion protein. However, the immunoreactive band (~120 kDa) was not detected in the control cells carrying the empty vector pVLT33. These results demonstrated the successful synthesis of INPNC-CH fusion protein in the recombinant strain YC-1 carrying pVIC3. To demonstrate the localization of INPNC-CH fusion protein in the outer membrane, the outer membrane and soluble fractions collected from cells carrying pVIC3 were probed with polyclonal anti-CH antibodies. As expected, the vast majority of INPNC-CH fusion proteins (accounting for ~90% of total amount) were associated with the outer membrane fraction as judged by the intensity of the protein bands (Fig. 1A, lane 4). To evaluate the quantity of CH displayed on the cell surface, CH activity was determined using whole cell, the outer membrane and total cell lysate. Over 90% of CH activity was detected in the outer membrane fraction of cells carrying pVIC3. In parallel, more than 90% of CH activity was present on the cell surface as judged by the ratio of whole-cell activity (0.241 U/OD_600_) to cell lysate activity (0.267 U/OD_600_).

Since proteinases can hardly penetrate the outer membrane, only proteins displayed on the cell surface can be degraded by proteinases^19,25^. In this study, the surface localization of INPNC-CH was also confirmed by proteinase accessibility experiment. After treatment with proteinase K for 3 h, whole-cell CH activity of the recombinant strain YC-1 carrying pVIC3 was decreased by 90% relative to proteinase untreated cells, which contrasted with the slight reduction (7%) for YC-1/pVC3 cells expressing cytosolic CH. The outer membrane fraction from YC-1/pVIC3 cells treated with proteinase K for 3 or 2.5 h was probed with polyclonal anti-CH antibodies. As expected, only ~10% or ~20% of INPNC-CH fusion proteins (relative to proteinase untreated cells) were detected in the outer membrane fraction as judged by the intensity of the protein bands because the vast majority of surface-displayed CH molecules were degraded by proteinase K (Fig. S1).

Immunolabeling with specific antibodies is a useful tool to detect proteins displayed on cell surface^19,42^. To confirm the presence of INPNC-CH on the cell surface of *Stenotrophomonas*, cells were probed with rabbit polyclonal anti-CH antibodies and then fluorescently stained with tetramethylrhodamine isothiocyanate (TRITC)-labeled goat anti-rabbit IgG antibody. Since antibodies cannot diffuse through the outer membrane, only surface-exposed CH can interact with its specific antibodies. Under a fluorescence microscope, YC-1/pVIC3 cells with surface-displayed CH were brightly fluorescent, whereas YC-1/pVC3 cells expressing cytosolic CH were not immunostained (data not shown), indicating that the cell surface of *Stenotrophomonas* was covered with antibody-TRITC complex.

After treatment with proteinase K for 3.5 h, cells carrying pVIC3 were probed with polyclonal anti-CH antibodies and TRITC-conjugated IgG secondary antibody. However, cells were not immunostained when observed with a fluorescence microscope, which indicated that surface-exposed CH had been removed by proteinase K. From all of these results, we concluded that CH was displayed functionally on the cell surface of *Stenotrophomonas* using the truncated InaV as an anchoring motif.

### CH activity and cell growth

**Figure 2:**
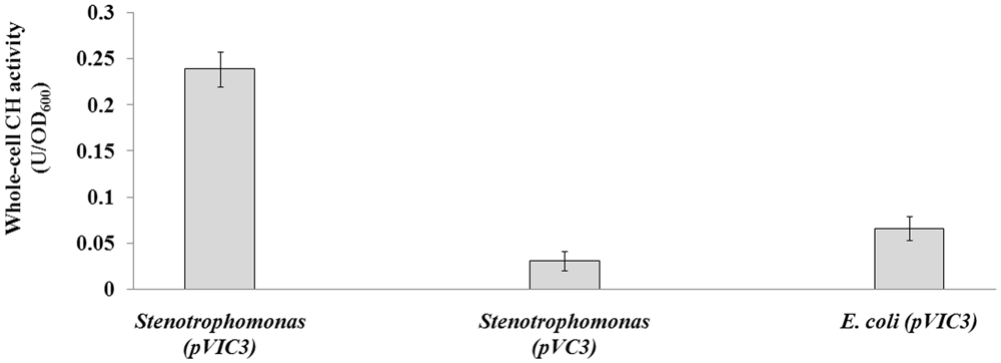
Whole-cell CH activity of *Stenotrophomonas* sp. strain YC-1 carrying pVC3 or pVIC3 and *E. coli* DH5α carrying pVIC3. The data are mean values ± standard deviations of three replicates.

Whole-cell CH activity of the recombinant *Stenotrophomonas* with surface-displayed CH (pVIC3) was 7.7-fold higher than that of the same strain with cytosolic CH (pVC3) (Fig. 2). This improvement in whole-cell CH activity may be attributed to the fact that the binding of CH to substrates is reinforced by the presentation of CH on cell surface. The recombinant strain with surface-displayed CH overcomes the mass transport limitation, and therefore it can be employed as a whole-cell biocatalyst for the hydrolysis of carbaryl. Moreover, the resting-cell suspension of the recombinant strain YC-1 with surface-displayed CH maintained the original CH activity during a 14-day incubation period (Fig. S2), suggesting that the enzyme is immobilized with enhanced structural stability on the outer cell membrane through the INP anchoring motif.

In this study, the effects of host strains on the efficiency of surface expression were also investigated. Whole-cell CH activity of the recombinant *Stenotrophomonas* carrying pVIC3 was 3.6-fold higher than that of *E. coli* DH5α carrying the same plasmid (Fig. 2). Subcellular fractionated samples of *E. coli* DH5α carrying pVIC3 were probed with polyclonal anti-CH antibodies. As a result, ~25% and ~75% of INPNC-CH fusion proteins were associated with the outer membrane and soluble fractions of DH5α/pVIC3 cells, respectively, as judged by the intensity of the protein bands (Fig. 1B), consistent with the activity distribution between the outer membrane (0.069 U/OD_600_) and soluble fractions (0.202 U/OD_600_). This high-level surface expression of CH in *Stenotrophomonas* is in accordance with previous report in which the INP system was used for cell surface display of OPH in *Stenotrophomonas* ^42^. Our results suggest that improved membrane translocation may occur with the INP system in *Stenotrophomonas*. This may be attributed to compatibility of the INP anchoring motif with the membrane structure of *Stenotrophomonas* since INP was originally isolated from strains of the genus *Pseudomonas* that has a close phylogenetic relationship with *Stenotrophomonas* ^43^. This study highlights the potential of *Stenotrophomonas* to be used as host strain for INP-mediated cell surface display.

**Figure 3:**
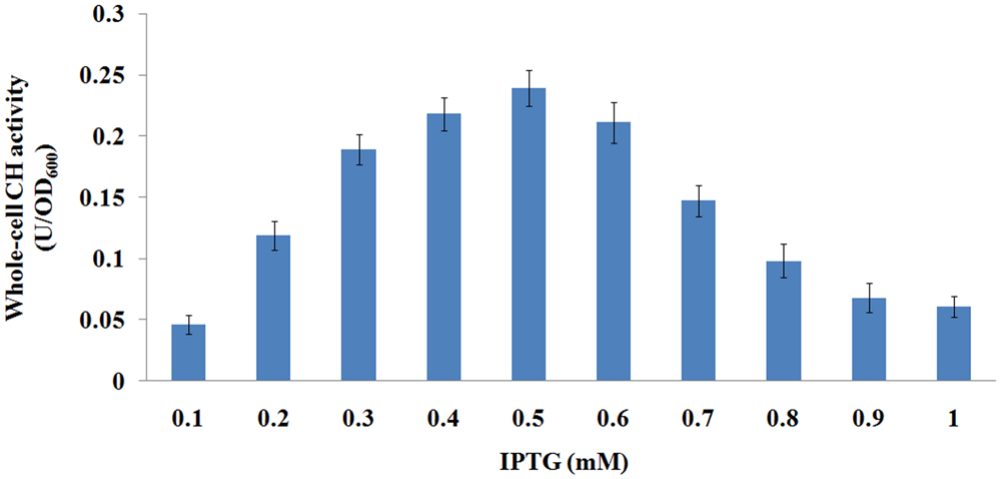
Whole-cell CH activity of *Stenotrophomonas* sp. strain YC-1 carrying pVIC3 under different levels of induction. The data are mean values ± standard deviations of three replicates.

Prior to IPTG induction, CH activity was not detected. Whole-cell CH activity increased gradually after induction with 0.5 mM IPTG and reached a maximum (0.24 U/OD_600_) at 24 h. Effects of different levels of induction on whole-cell CH activity were also investigated. Whole-cell CH activity increased gradually with increasing concentrations of IPTG and reached a maximum (0.239 U/OD_600_) at an IPTG concentration of 0.5 mM. However, induction with higher IPTG concentrations (0.6 to 1 mM) caused declines in whole-cell CH activity (Fig. 3), probably because a high transcription rate can block the translocation pathway of a secreted protein. The inhibitory effects of overexpression on protein translocation have been reported in previous studies on INP-mediated cell surface display of GFP and OPH^20,25^. Here, the optimal balance between protein expression and translocation was accomplished by the use of an inducible expression system.

**Figure 4:**
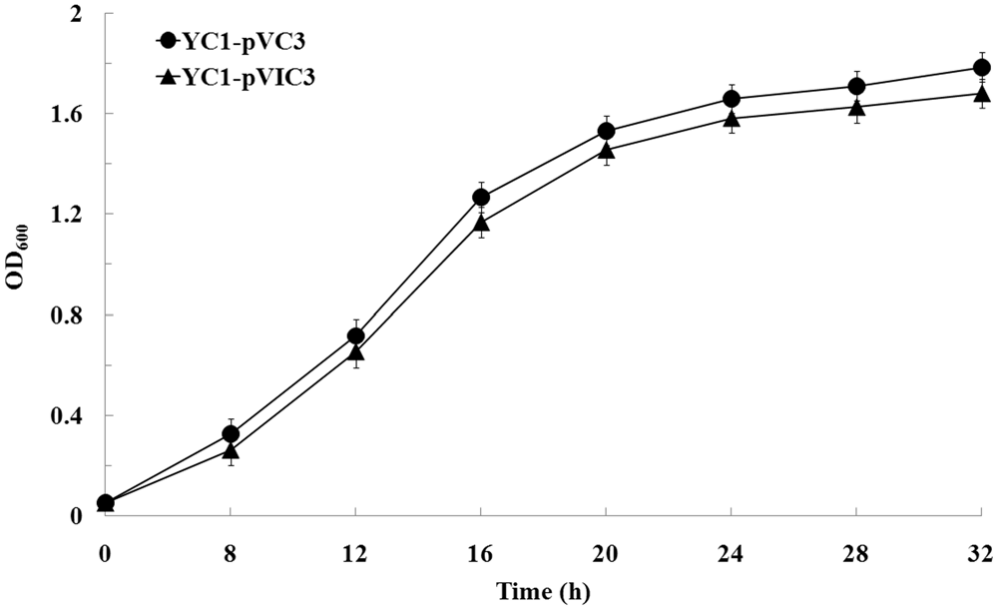
Cell growth curves of the recombinant *Stenotrophomonas* sp. strains YC-1/pVC3 and YC-1/pVIC3. Cells were incubated in LB medium supplemented with 50 μg/mL kanamycin and 0.5 mM IPTG at 30 °C for 32 h. The data are mean values ± standard deviations of three replicates.

Display of macromolecular heterologous proteins on the bacterial cell surface may result in instability of the outer membrane and growth inhibition of the cell^21,22^. To test whether surface display of CH inhibits cell growth, the growth kinetics of YC-1/pVIC3 and YC-1/pVC3 cells were compared. As expected, no growth inhibition was observed for YC-1/pVIC3 cells. YC-1/pVIC3 and YC-1/pVC3 cells showed similar growth profiles and reached a maximum OD_600_ of 1.681 and 1.787 at 32 h, respectively (Fig. 4). These results indicated that surface display of CH did not disturb the membrane structure or cause host growth defects. In previous studies, the INP-based display systems have been used to display several large heterologous proteins, such as 90 kDa chitinase^28^ and 77 kDa CPR^29^, on the cell surface of *E. coli*. In this study, we successfully expressed 88 kDa CH on the *Stenotrophomonas* cell surface using the truncated InaV as an anchoring motif, suggesting that the INP system is well suited as a carrier of relatively large inserts for functional expression of macromolecular foreign proteins on the surface of bacterial cell. To our knowledge, CH is the largest protein displayed with the INP system in bacteria other than *E. coli* so far.

### Simultaneous hydrolysis of carbaryl and chlorpyrifos by the recombinant strain YC-1

**Figure 5:**
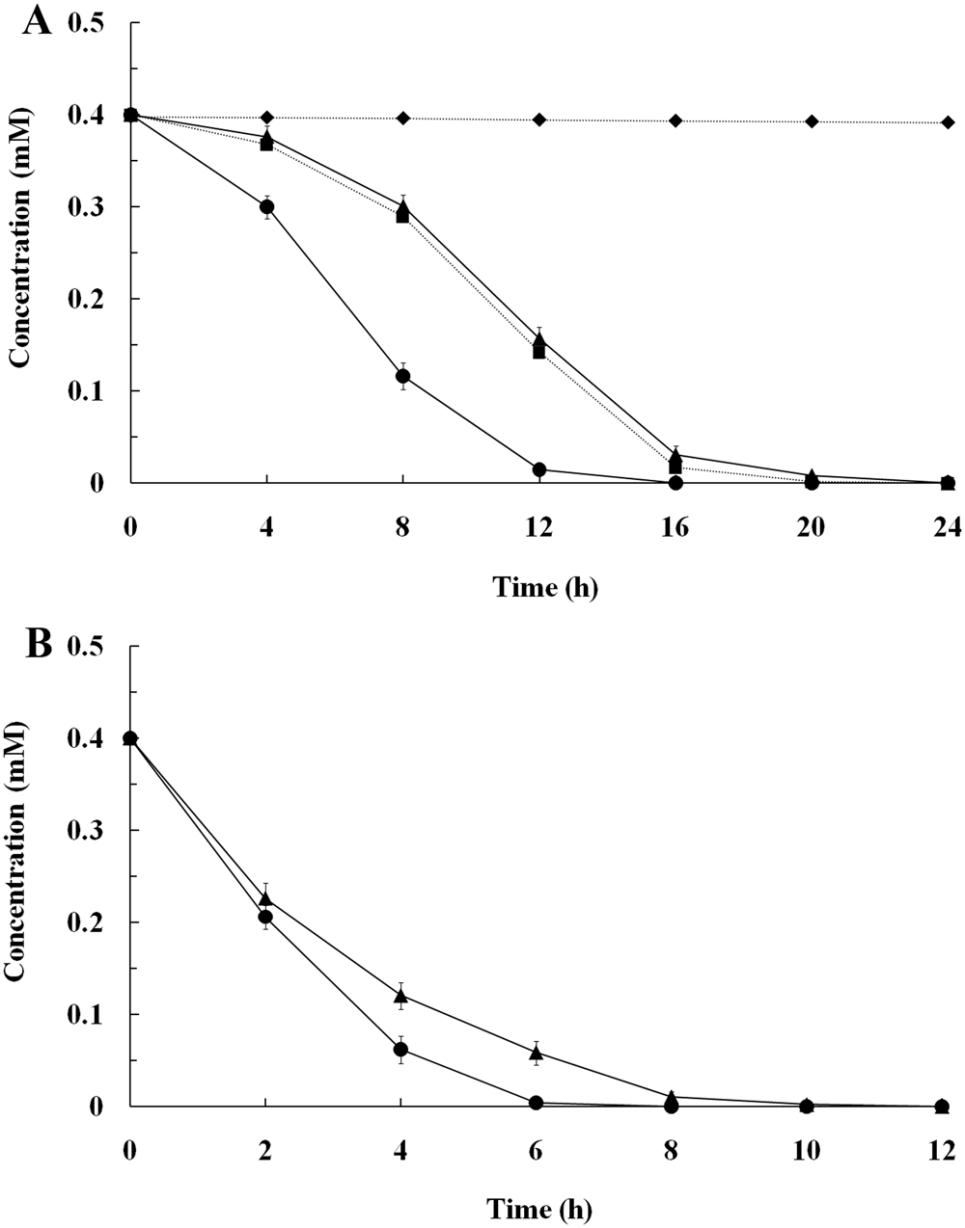
(**A**) Hydrolysis of carbaryl and chlorpyrifos by growing cells (with an initial cell density of OD_600_ = 0.1) of the wild-type and recombinant strain YC-1. (**B**) Hydrolysis of carbaryl and chlorpyrifos by resting-cell suspension (OD_600_ = 1.0) of the recombinant strain YC-1. Symbols: ▲, hydrolysis of chlorpyrifos by YC-1/pVIC3 cell; ■, hydrolysis of chlorpyrifos by YC-1 cell; ●, hydrolysis of carbaryl by YC-1/pVIC3 cell; ♦, hydrolysis of carbaryl by YC-1 cell. The data are mean values ± standard deviations of three replicates.

In order to evaluate the pesticide hydrolysis capability of the recombinant strain YC-1 with surface-displayed CH, the hydrolysis experiments for carbaryl and chlorpyrifos were carried out with growing and resting cells of YC-1/pVIC3. As shown in Fig. 5A, 0.4 mM carbaryl and chlorpyrifos were completely hydrolyzed by growing cells (with an initial cell density of OD_600_ = 0.1) of YC-1/pVIC3 within 16 and 24 h, respectively. Moreover, 0.4 mM carbaryl and chlorpyrifos were completely hydrolyzed by resting-cell suspension (OD_600_ = 1.0) of YC-1/pVIC3 within 8 and 12 h, respectively (Fig. 5B). As expected, carbaryl could not be hydrolyzed by wild-type strain YC-1 (Fig. 5A), indicating that the recombinant strain YC-1 acquired the hydrolysis capability for carbaryl through the heterologous expression of CH. The recombinant strain YC-1 hydrolyzed chlorpyrifos as fast as wild-type strain YC-1 (Fig. 5A), indicating that surface expression of CH did not influence the intrinsic hydrolysis capability of strain YC-1 for chlorpyrifos. As expected, the concentration of carbaryl and chlorpyrifos did not change in non-inoculated control. These results suggest that the recombinant strain YC-1 can be employed for simultaneous hydrolysis of carbaryl and chlorpyrifos.

The recombinant strain YC-1 with endogenous CPH can transform chlorpyrifos to 3,5,6-trichloro-2-pyridinol (TCP) and diethylthiophosphoric acid (DETP) and utilize DETP as a carbon source, but TCP cannot be further degraded. The recombinant strain YC-1 with exogenous CH can hydrolyze carbaryl to 1-naphthol and methylamine, but the products cannot be further metabolized due to lack of other carbaryl-degrading genes.

## Conclusions

Practical applications of large-scale enzymatic degradation have always been limited by the cost of purification and stability of the enzyme. The use of whole-cell biocatalysts is an alternative strategy for degradation of toxic pollutants. In this work, a whole-cell biocatalyst was developed for efficient hydrolysis of carbaryl and chlorpyrifos by the display of CH on the cell surface of a chlorpyrifos-degrading bacterium using an INP-based surface-anchoring system. Owing to its high activity and superior stability, the live biocatalyst is ideal for the decontamination of sites polluted with pesticides. Moreover, our findings highlight the potential of the INP system for the display of large proteins on cell surfaces of various environmental bacteria, and suggest that the species *Stenotrophomonas* may serve as a promising candidate for the development of versatile whole-cell biocatalysis systems by the cell surface display of a wide variety of novel enzymes.

## Materials and Methods

### Bacterial strains, plasmids, and culture conditions

A chlorpyrifos-degrading bacterium *Stenotrophomonas* sp. strain YC-1, which was isolated from activated sludge of the wastewater treating system of an organophosphorus pesticides manufacturer^13^, was used as host strain for cell surface display of CH. A surface expression vector, pVIC3, coding for INPNC-CH was used for targeting CH onto the cell surface of strain YC-1. An *inpnc-cehA* fusion gene was chemically synthesized by BGI Inc., Beijing, China and then ligated into pUC57-simple vector. The synthetic gene was released from pUC57-simple with *Eco*RI and *Hin*dIII and then subcloned into the same restriction sites of pVLT33, an *Escherichia coli*-*Pseudomonas* shuttle vector, to generate pVIC3. To construct a control plasmid expressing cytosolic CH, the *cehA* gene was amplified by PCR from pVIC3, digested with *Eco*RI and *Hin*dIII, and subcloned into the same restriction sites of pVLT33 to generate pVC3.

Strains bearing plasmids were grown in Luria-Bertani (LB) medium^44^ or M9 minimal medium^45^ supplemented with 50 μg/ml kanamycin at 30 °C. Transformation of plasmid into *Stenotrophomonas* was carried out using the electroporation method^46^. For the inducible expression of INPNC-CH fusion protein, 0.5 mM IPTG was added when the culture reached an OD_600_ of 0.4. After induction, the cultivation continued for additional 24 h at 30 °C.

### Western blot analysis

Cells carrying pVIC3 were divided into total cell lysate and the outer membrane and soluble fractions by differential centrifugation^19^. Subcellular fractionated samples were analyzed by 12% SDS-PAGE^44^, after which protein samples were electroblotted using a semidry transfer system (Bio-Rad) onto a PVDF membrane (Roche). The membrane was blocked for 1 h in TBST (20 mM Tris-HCl, pH 7.5, 150 mM NaCl, 0.05% Tween 20) containing 3% BSA. Subsequently, the membrane was incubated with rabbit polyclonal anti-CH antibodies at a dilution of 1:1,000 for 2 h, after which the membrane was washed with TBST for 30 min and then incubated with a horseradish peroxidase-conjugated goat anti-rabbit IgG secondary antibody (Abcam) at a dilution of 1:2,000 for 1.5 h. Immunoreactive bands were detected by enhanced chemiluminescence using an ECL Plus kit (Amersham).

### Immunofluorescence microscope

Cells carrying pVIC3 or pVC3 were resuspended (OD_600_ = 0.5) in 100 mM phosphate-buffered saline (PBS; pH 7.4) containing 3% BSA and then incubated with rabbit polyclonal anti-CH antibodies diluted (1:500) in PBS for 1.5 h at room temperature. After being washed with PBS, the cells were resuspended in PBS with goat anti-rabbit IgG antibody conjugated with TRITC (1:2,000 dilution, Invitrogen) and incubated for 1 h at room temperature. Prior to microscopic observation, cells were washed five times with PBS and mounted on poly(L-lysine)-coated microscopic slides. Photographs were taken using a Nikon fluorescence microscope equipped with TRITC and FITC filters.

### CH activity assay

CH activity was assayed in a reaction mixture containing 0.5 mM carbaryl, 50 mM sodium phosphate buffer (pH 7.0), and 100 μl of cells (OD_600_ = 1.0) in a total volume of 1.0 ml. The reaction was started by adding the cells and incubated at 30 °C. The reaction was stopped by adding 100 μl of 2 mM HgCl_2_, and 1-naphthol produced was quantified by reacting with the fast blue B salt using colorimetric analysis^6^. Activities are expressed as units (1 μmol of 1-naphthol formed per minute at 30 °C) per OD_600_ of whole cells.

### Proteinase accessibility experiment

Cells carrying pVIC3 or pVC3 were resuspended in 1 ml of 15 mM Tris-HCl buffer (pH 7.8) supplemented with 15% sucrose and 0.1 mM EDTA. Samples were incubated for 2.5, 3 or 3.5 h with 5 μl of 20 mg/ml proteinase K at room temperature. Proteinase treated and untreated cells were used for CH activity assay, Western blot analysis, and immunofluorescence microscope as described above.

### Pesticide hydrolysis studies

YC-1/pVIC3 cells were harvested after incubation in LB medium plus kanamycin and resuspended (OD_600_ = 1.0) in M9 minimal medium. Subsequently, 10 ml of cell suspension was inoculated into 90 ml of M9 minimal medium supplemented with 0.2% glucose, 0.05% yeast extract, 50 μg/ml kanamycin, 0.5 mM IPTG, and 0.4 mM carbaryl and chlorpyrifos. Samples were incubated at 30 °C and 200 rpm and removed every 4 h for the quantitative analysis of carbaryl and chlorpyrifos using high performance liquid chromatography (HPLC). Pesticide extraction and HPLC analysis were carried out as described previously^45^.

YC-1/pVIC3 cells were inoculated at OD_600_ = 0.1 into M9 minimal medium supplemented with 0.2% glucose, 0.05% yeast extract, 50 μg/ml kanamycin, 0.5 mM IPTG, and 0.2 mM chlorpyrifos. After incubation for 24 h at 30 °C, cells were harvested, washed twice with 50 mM sodium phosphate buffer (pH 7.0), and resuspended (OD_600_ = 1.0) in the same buffer. Subsequently, the cell suspension was incubated with 0.4 mM carbaryl and chlorpyrifos at 200 rpm and 30 °C. The samples were withdrawn every 2 h and carbaryl and chlorpyrifos were quantified by HPLC.

## Electronic supplementary material

Supplemental material
