# Supplementary material for: Simultaneous hydrolysis of carbaryl and chlorpyrifos by Stenotrophomonas sp. strain YC-1 with surface-displayed carbaryl hydrolase
Source: Sci Rep. 2017 Oct 17;7:13391. doi: 10.1038/s41598-017-13788-0 (PMC5645314; doi:10.1038/s41598-017-13788-0)
Supplement: Supplementary file 1 — Supplemental material [file 41598_2017_13788_MOESM1_ESM.pdf]

Supplemental material

Simultaneous hydrolysis of carbaryl and chlorpyrifos by *Stenotrophomonas* sp. strain YC-1 with surface-displayed carbaryl hydrolase

Chao Yang,[1] Xiaoqing Xu,[1] Yanping Liu,[2*] Hong Jiang,[3] Yunbo Wu,[1] Ping Xu,[4] Ruihua Liu[1*]

[1]College of Life Sciences, Nankai University, Tianjin 300071, China

[2]Department of Gynaecology and Obstetrics, Tianjin Medical University General Hospital, Tianjin 300052, China

[3]Institute of Zoology, Chinese Academy of Sciences, Beijing 100101, China

[4]State Key Laboratory of Microbial Metabolism, School of Life Sciences and Biotechnology, Shanghai Jiao Tong University, Shanghai 200240, China

*Corresponding authors:

Yanping Liu (Tel.: 86 22 6036 2960; Fax: 86 22 6036 3150; E-mail: lyp5588@sohu.com)

Ruihua Liu (Tel./ Fax: 86 22 2350 2351; E-mail: yangyangliu@nankai.edu.cn)

Chao Yang and Xiaoqing Xu contributed equally to this work.

Fig. S1. Western blot analysis for quantifying INPNC-CH fusion protein in the outer membrane fraction from *Stenotrophomonas* sp. strain YC-1/pVIC3 cells with or without proteinase K treatment.

Fig. S2. Whole-cell CH activity in resting cultures of the recombinant *Stenotrophomonas* sp. strain YC-1 with surface-expressed CH.

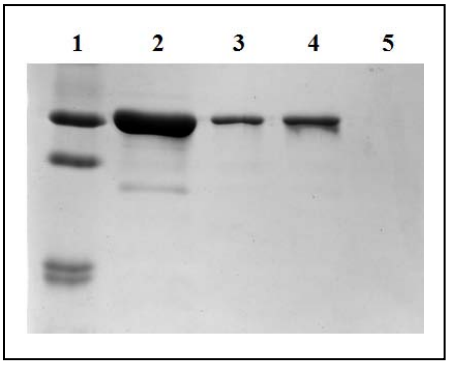

Fig. S1. Western blot analysis for quantifying INPNC-CH fusion protein in the outer membrane fraction from *Stenotrophomonas* sp. strain YC-1/pVIC3 cells with or without proteinase K treatment. Lane 1, protein marker; lane 2, outer membrane fraction from proteinase K untreated cells; lane 3, outer membrane fraction from cells treated with proteinase K for 3 h; lane 4, outer membrane fraction from cells treated with proteinase K for 2.5 h; lane 5, whole-cell lysates of YC-1/pVLT33.

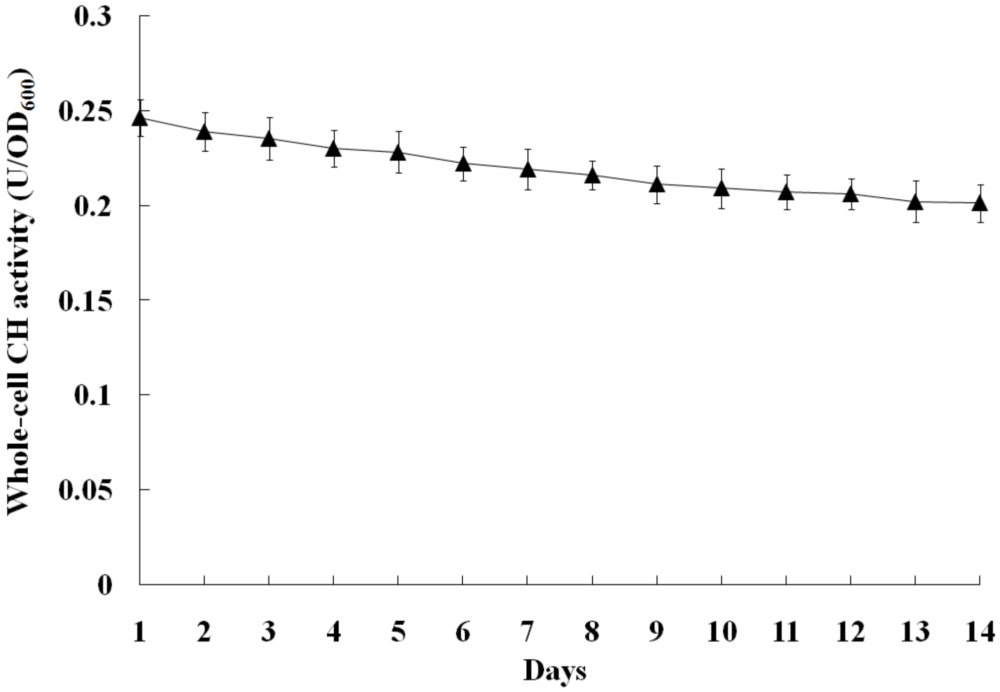

Fig. S2. Whole-cell CH activity in resting cultures of the recombinant *Stenotrophomonas* sp. strain YC-1 with surface-expressed CH. YC-1/pVIC3 cells were grown in 50 ml of LB medium supplemented with 0.5 mM IPTG and 50 μg/ml kanamycin for 24 h. Collected cells were resuspended in 5 ml of 50 mM sodium phosphate buffer (pH 7.0) and cell suspensions were then incubated with shaking at 30°C. Over a 2-week period, 0.1 ml of samples was removed each day. Samples were centrifuged and resuspended in 0.1 ml of 50 mM sodium phosphate buffer (pH 7.0). CH activity assays were conducted as described in Materials and methods. The data are mean values ± standard deviations of three replicates.

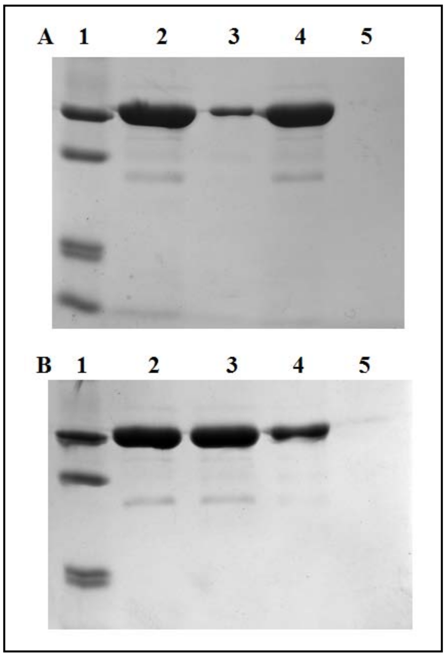

Fig. 1. Western blot analysis for subcellular localization of INPNC-CH fusion protein in *Stenotrophomonas* sp. strain YC-1/pVIC3 (A) and *E. coli* DH5α/pVIC3 (B). Lane 1, protein marker; lane 2, whole-cell lysates; lane 3, soluble fraction; lane 4, outer membrane fraction; lane 5, whole-cell lysates of YC-1/pVLT33 (or DH5α/pVLT33).
